# Therapeutic Potential of Endothelial Progenitor Cells in Angiogenesis and Cardiac Regeneration: A Systematic Review and Meta-Analysis of Rodent Models

**DOI:** 10.34172/apb.025.45122

**Published:** 2025-06-16

**Authors:** Samaneh Narimani, Reza Rahbarghazi, Hanieh Salehipourmehr, Maryam Taghavi Narmi, Hamid Lotfimehr, Robab Mehdipour

**Affiliations:** ^1^Department of Applied Cell Sciences, Faculty of Advanced Medical Sciences, Tabriz University of Medical Sciences, Tabriz, Iran; ^2^Stem Cell Research Center, Tabriz University of Medical Sciences, Tabriz, Iran; ^3^Research Center for Evidence-Based Medicine, Tabriz University of Medical Sciences, Tabriz, Iran

**Keywords:** Endothelial progenitor cells, Myocardial infarction, Rodents, Regenerative outcomes

## Abstract

**Purpose:**

Myocardial infarction (MI), the leading cause of human mortality, is induced by a sudden interruption of blood supply. Among various stem cell types, endothelial progenitor cells (EPCs) are novel and valid cell sources for the restoration of vascularization in the ischemic tissue. The present study aimed to evaluate the regenerative properties of EPCs in rodent models of MI.

**Methods:**

A comprehensive systematic search was implemented in Cochrane Library, Embase, PubMed, Scopus, and Web of Science databases without language limitation in Sep 2024. Of the 67 papers pooled, 42 met the inclusion criteria and were subjected to multiple analyses.

**Results:**

Compared to the MI group, the overall effect size was confirmed in the groups receiving EPC with enhanced angiogenesis (SMD: 2.02, CI 95%: 1.51-2.54, *P*<0.00001; I^2^: 82%), reduced fibrosis (SMD: -1.48; 95% CI−2.15, -0.81; *P*<0.0001; I^2^: 88%), improved ejection fraction (EF; SMD: 1.72; 95% CI−1.21, 2.23; *P*<0.00001; I^2^: 87%), and fractional shortening (FS; SMD: 1.58; 95% CI−1.13, 2.03; *P*<0.00001; I^2^: 82%). Data confirmed significant improvements in the cardiac tissue parameters after intramyocardial injection of EPCs.

**Conclusion:**

These data showed that EPC transplantation is an alternative therapy to ameliorate ischemic myocardium in rodents via the stimulation of angiogenesis, reduction of fibrosis, and improvement of fractional shortening and ejection fraction.

## Introduction

 Ischemic heart disease (IHD) is a global leading cause of human mortality and disability in the clinical setting.^1^ Typically, myocardial infarction (MI) occurs following partial or complete occlusion of a coronary artery leading to massive cardiomyocyte damage, inflammation, and subsequent fibrotic changes.^2^ Notably, the contraction of fibroblasts and collagen fibers at the healing site can contribute to the thinning of the left ventricle (LV). Over time, the reduction of ejection fraction (EF) and lethal arrhythmias in an ischemic heart can be life-threatening.^3^ Currently, percutaneous coronary intervention (PCI) and coronary artery bypass grafting (CABG) are clinical modalities for the restoration of blood and reduction of cardiomyocyte injury.^4^ Unfortunately, these approaches are not fully effective, and the development and application of *de novo* therapeutic strategies are highly recommended.^5^

 In recent decades, the discovery and application of stem cells in various pathological conditions have revolutionized regenerative medicine.^6^ It has been shown that stem cells can promote the healing of ischemic myocardium via the release of cytokines, growth factors, and direct differentiation into cardiomyocytes.^6,7^ Besides, these cells can accelerate the regeneration of injured myocardium via juxtacrine interaction and production of pro-angiogenesis factors.^8,9^

 According to recent data, it has been confirmed that endothelial progenitor cells (EPCs) are valid cell sources for restoring dysfunctional endothelium via various reparative functions, especially promoting angiogenesis and vasculogenesis.^10^ In this regard, EPCs alone or in combination with other stem cells or mature cells have been used in different studies to accelerate regenerative outcomes and circumvent limitations associated with the administration of single stem cell type alone.^11,12^ Proteomic analyses have proved the existence of common specific surface molecules such as CD133, CD34, vascular endothelial growth factor-2 (VEGFR-2), Tie-2, and Sca-1 between EPCs and hematopoietic stem cells.^13^ Following various pathologies and hypoxic conditions, EPCs are recruited from the bone marrow niche, the primary storage site in adults, to the circulation system.^14^ Circulating EPCs migrate toward the injury sites in a cytokine gradient manner where they gradually lose their stemness features (CD133↓, and CD34↓) and mature into endothelial cells (ECs; CD31↑ and vWF↑).^15^ Besides differentiation capacity, EPCs release several proangiogenesis factors (IGF-1, VEGF, HGF, FGF-2, etc.) to expedite the formation of new blood vessels in the hypoxic areas.^16^ Data have indicated that the injection of EPCs in several animal models of MI can improve the healing of myocardium through the stimulation of angiogenesis, regulation of inflammation, and control of extracellular matrix (ECM) remodeling.^12^

 In the present systematic review, the application of EPCs in the rodent model of MI and their potential in the restoration of injured myocardium mainly via angiogenesis was explored. To the best of our knowledge, there are few reports related to systematic review and metanalysis of EPCs in humans and different animal models of MI. Most of the studies have investigated the diagnostic properties of EPCs under certain pathological conditions such as ischemic diseases in humans or there are several reposts related to separate applications of EPCs in certain MI models in animals.^17-19^ Although the reparative properties of EPCs have been proved in different MI animal models, it is imperative that data from various experiments with similar objectives be combined and assessed to minimize the possible bias and make logic in the interpretation of the obtained data.^20^ In the last decades, rodents have been widely used for different experiments related to the MI model due to inherent advantages like small body mass and easy handling pre- and post-MI induction with minimal space and resources. Besides, researchers can have access to various rodents with similar genetic characteristics which facilitates high repeatability.^21^ It seems that data from this study can provide invaluable data about the eligibility of EPC application in the alleviation of MI in the clinical setting.

## Material and Methods

 The current systematic review and meta-analysis were conducted based on the PRISMA 2020 statement guideline. The used protocol was registered in the PROSPERO database (CRD42024571517).

###  Search strategy

 A comprehensive systematic search was implemented in Cochrane Library, Embase, PubMed, Scopus, and Web of Science databases without the limitations of language and date in Sep 2024. After the completion of the systematic search, collected articles, experiments, and contacted authors were carefully monitored and validated for subsequent evaluations. The abstracts from the international congresses were also monitored. The strategy used in this study is shown in Table S1 (see Supplementary file 1).

###  Study design considerations

 All preclinical studies associated with the application of EPCs in rodent models of MI, including mice and rats were reviewed. Rodents with experimentally induced MI in any age in both genders were included. EPCs transplantation in human counterparts, and other species (*i.e.*, rabbits, porcine, canines, etc.), and *in vitro* experiments were excluded from the present analysis. Data related to the administration of EPCs alone, but not in combination with other stem cell types, were collected. Also, studies related to the use of EPC exosomes in rodent models of MI were not included. Articles with no access to their full texts were not considered. In Table 1, inclusion and exclusion criteria are outlined.

**Table 1:** Inclusion and exclusion criteria

**Inclusion criteria**	**Exclusion criteria**
Preclinical studies about EPCs as therapy on rodent models (mice and rats), with cardiac infarction in any age or gender Endothelial progenitor cells Studies including the combination therapy with EPC such as scaffold, miRNA, growth factors, and other type of stem cells Studies with CD34 + cells transplantation All experimental studies (preclinical)	Not an animal study Other animal study rather than rodents Not a myocardial infarction model Clinical studies on humans In vitro studies Other types of stem cells Studies with CD133 + cells transplantation Studies including combination therapy with EPC and other types of stem cells Not transplantation of EPC and just mobilization investigation EPCs-derived exosome transplantation Other study type In vitro studies Studies without any access to the full text, or studies in the other languages, and retracted studies

 The primary outcome indicators were “angiogenesis”, and “infarct size”. The secondary outcome indicators were “LVEF”, and “fractional shortening (FS)”. For the meta-analysis, the data containing at least one of the outcomes measured between 1- and 8 weeks post-EPC transplantation were used. If studies contained more than one set of data for primary or secondary outcome analysis, the selection was done based on the more relevant and common data.

###  Study selection

 Once the databases were searched for the relevant papers, all collected citations were uploaded to EndNote 18 software with duplicate studies being deleted. Two separate reviewers blindly screened the titles and abstracts to ensure the eligibility of the studies in terms of the inclusion and exclusion criteria. Any discrepancy was re-checked again by a third blind reviewer.

###  Data collection

 The collected data from multiple search databases were organized using PRISMA guidelines. For this purpose, articles were entered into an Excel spreadsheet. The process was continued by an independent review of the selected abstracts by the same reviewers. Any disagreements were critically assessed until a precise decision was made and the opinion of a third reviewer was obtained if it was required.

###  Evaluation of methodological quality

 Using the modified CAMARADES checklist, two independent reviewers monitored the methodological validity of the quantitative publications selected for retrieval before their inclusion in the systematic review. Again, any disagreements were resolved through consultation with a third reviewer.

###  Statistical analysis

 The results of the selected data were analyzed using RevMan 5.4.1. Data are presented as mean ± SD with a 95% confidence interval (CI). Statistical heterogeneity was analyzed using the I^2^ value and the chi-square test. In this study, *P* < 0.05 and I^2^ > 50% were considered statistical heterogeneity. Fixed and mixed models were used for low and high heterogeneity in the parameters analysis. The subgroup analysis was performed if needed. Publication bias was assessed using funnel plots and more formally with both Begg and Mazumdar’s rank correlation test (Kendall’s tau) and Egger’s regression test. Begg’s test assesses the correlation between the effect estimates and their variances, while Egger’s test examines the relationship between the effect estimates and their standard errors. A *P* value of less than 0.05 was indicative of statistically significant publication bias.

## Results

###  Description of studies and risk of bias

**Figure 1:**
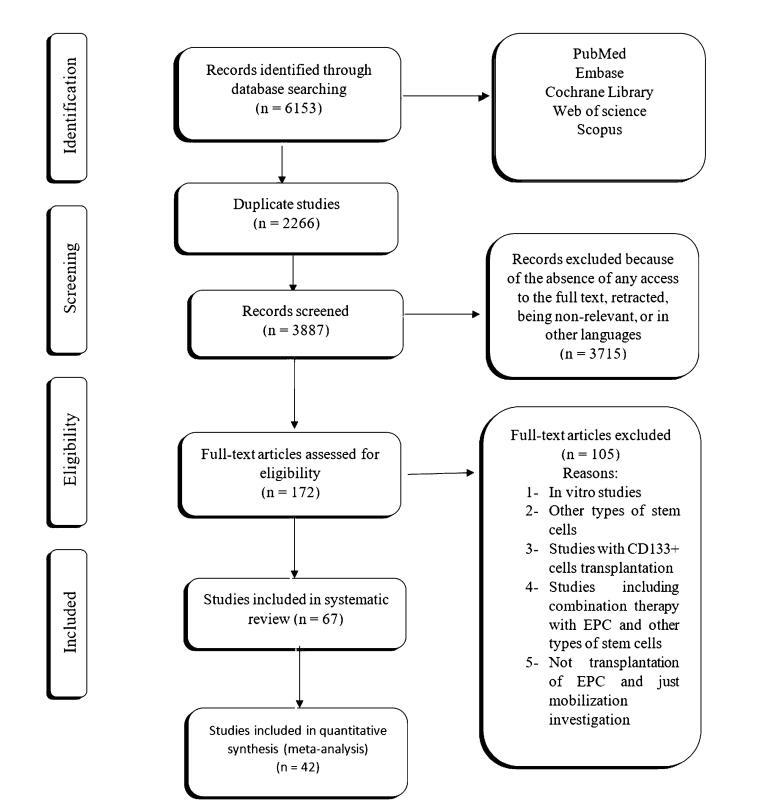
PRISMA diagram of the review process for the meta-analysis

**Figure 2:**
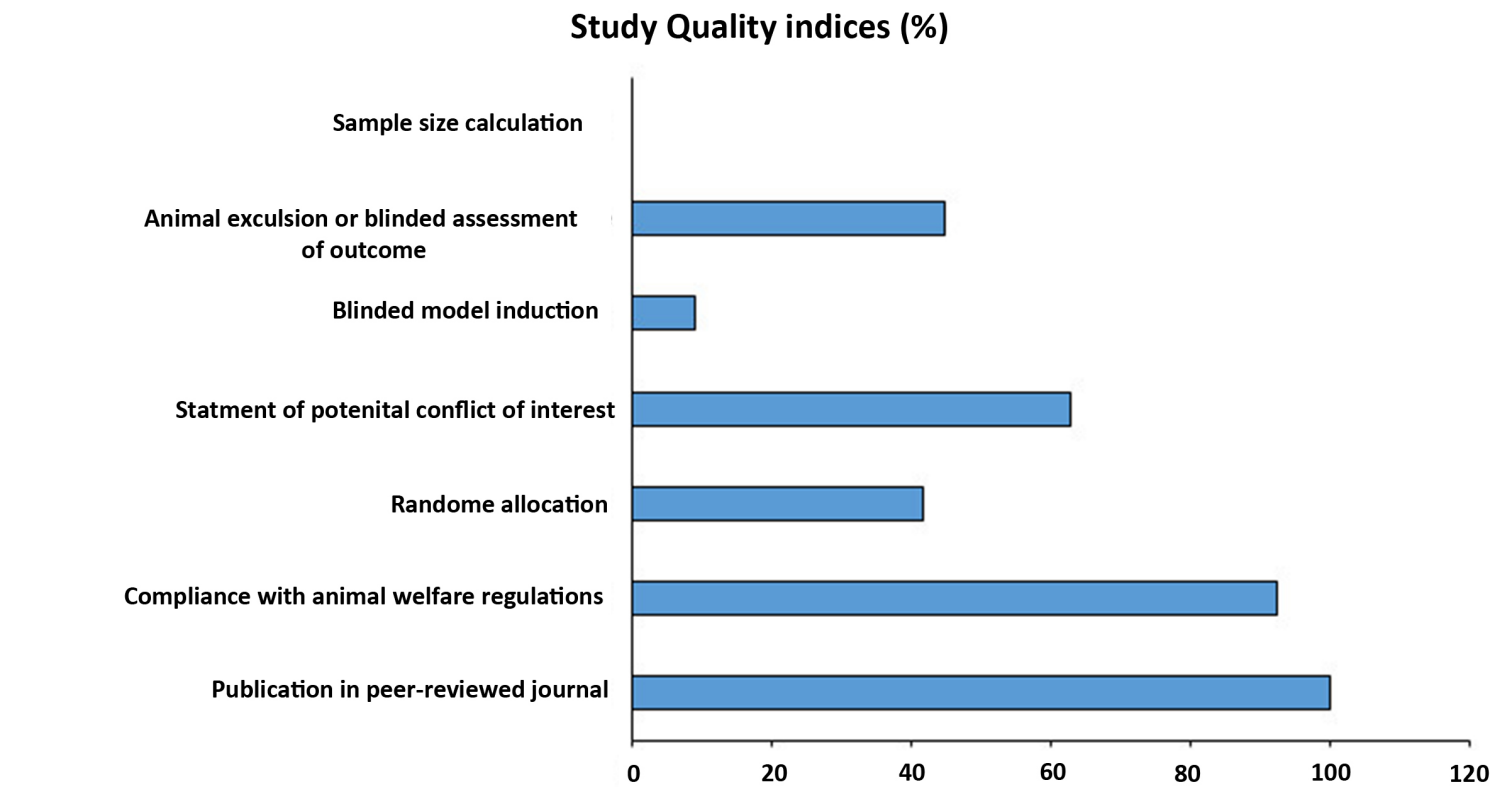
Percentage of selected experiments for each item in the modified version of the CAMARADES (Collaborative Approach to Meta-Analysis and Review of Animal Data from Experimental Studies) quality checklist

 The flow chart for data selection and handling is presented in Figure 1. Here, a modified CAMARADES quality checklist was used to assess the collected experiments. Of all peer-reviewed articles, 67 declared compliances with animal welfare regulations. It is worth mentioning that random allocation to different groups was detected in 28 studies and 42 experiments expressed a conflict-of-interest statement. Furthermore, 6 articles had blinded induction of MI in the rodent models and 30 studies benefitted from both animal exclusion criteria and blind outcome assessment based on our evaluation. In the selected articles, no study declared the methodology related to sample size calculation (Figure 2). All articles were included for quality synthesis (Table S2; See Supplementary file 2).

###  Characteristics of studies

 All the included studies from 2004 to 2021 with access to full text were selected. The systematic review focused on rodent models of MI consisting of rat (N = 37; 55.22%) and mouse (N = 30; 44.78%) models of MI (Table S2). Data indicated that a greater number of experiments were done on male rats/mice (N = 41; 61.19%), while 17 (25.37%) studies were conducted on female models Interestingly, in one study both genders were used. Rodents in 36 studies (53.73%) aged between 4 to 20 weeks. In 8 experiments (11.94%), the term “adult” was used to describe rodent age. In just one experiment (1.49%), “at least 9-week-old” rodents were used for the MI model. Rats and mice subjected to MI models weighed 80-350, and 18-250 grams, respectively. 58.21% of rats and mice were in healthy status (N = 39). Nude animals constituted 25.37% (N = 17) of the experiments. In 3 studies (4.48%), MI was inducted on diabetic models. Animals with severe combined immunodeficiency including NOD-SCID (N = 2; 2.99%), SCID (N = 1; 1.49%), and a combination of Nude/J or NOD-SCID (N = 1; 1.49%) were employed. Immunocompetent experimental models were 1.49% of collected studies (N = 1). In one study (1.49%), the models underwent ovariectomy together with splenectomy; while in one experiment just ovariectomy was conducted (1.49%). Experiments with both wild-type and IL-10 knockout models comprised 1.49% (N = 1) of the studies. Protocols consisting direct left anterior descending coronary artery (LAD) ligation (N = 64; 95.52%); injection of vitamin D3 in high-fat diet-fed rodents (N = 1; 1.49%), intramyocardial administration of microembolism suspension following the occlusion of the ascending aorta (N = 1; 1.49%), and LAD ligation followed by reperfusion besides aorta cross-clamping (N = 1; 1.49%) were used to induce experimental MI models. Based on the analysis, MI (N = 63; 94.03%), progressive MI to cardiomyopathy (N = 1; 1.49%), MI with ischemic reperfusion (N = 1; 1.49%), coronary artery microembolization (CME) (N = 1; 1.49%), and ICM (ischemic cardiomyopathy model) (N = 1; 1.49%) were pathological conditions in rodent models. In the selected articles, EPCs were collected from different sources as follows; Bone marrow (N = 32; 47.76%), peripheral blood (N = 19; 28.36%), umbilical cord blood (N = 11; 16.42%), direct cardio-puncture (N = 1; 1.49%), spleen (N = 1; 1.49%), dental pulp (N = 1; 1.49%), and both peripheral blood and bone marrow (N = 1; 1.49%). EPCs were administrated as doses between 2 × 10^2^ and 2 × 10^7^ in most of the experiments (N = 59; 88.06%). In contrast to studies using single EPC injection, 8 experiments (11.94%) were conducted based on multiple EPC administrations. Timing of EPC injection varied from immediate to delayed administration (until 4 weeks) following MI induction. Different introduction approaches and terms were found in different studies such as intramyocardial injection (N = 44; 65.67%), intravenous injection (N = 9; 13.43%), intramyocardial injection and subsequent treatment with the construct (N = 3; 4.48%), simultaneous intramyocardial and intracoronary injections (N = 2; 2.99%), injection into the LV (N = 2; 2.99%), transepicardial injection (N = 1; 1.49%), anterolateral LV surface suture (N = 1; 1.49%), implantation (N = 1; 1.49%), intracoronary injection (N = 1; 1.49%), percutaneously injection into LV (N = 1; 1.49%), injection to the border of occluded region (N = 1; 1.49%), and intramyocardial (intramuscular) or systemic injection (N = 1; 1.49%).

###  EPC transplantation effect on angiogenesis potential

**Figure 3:**
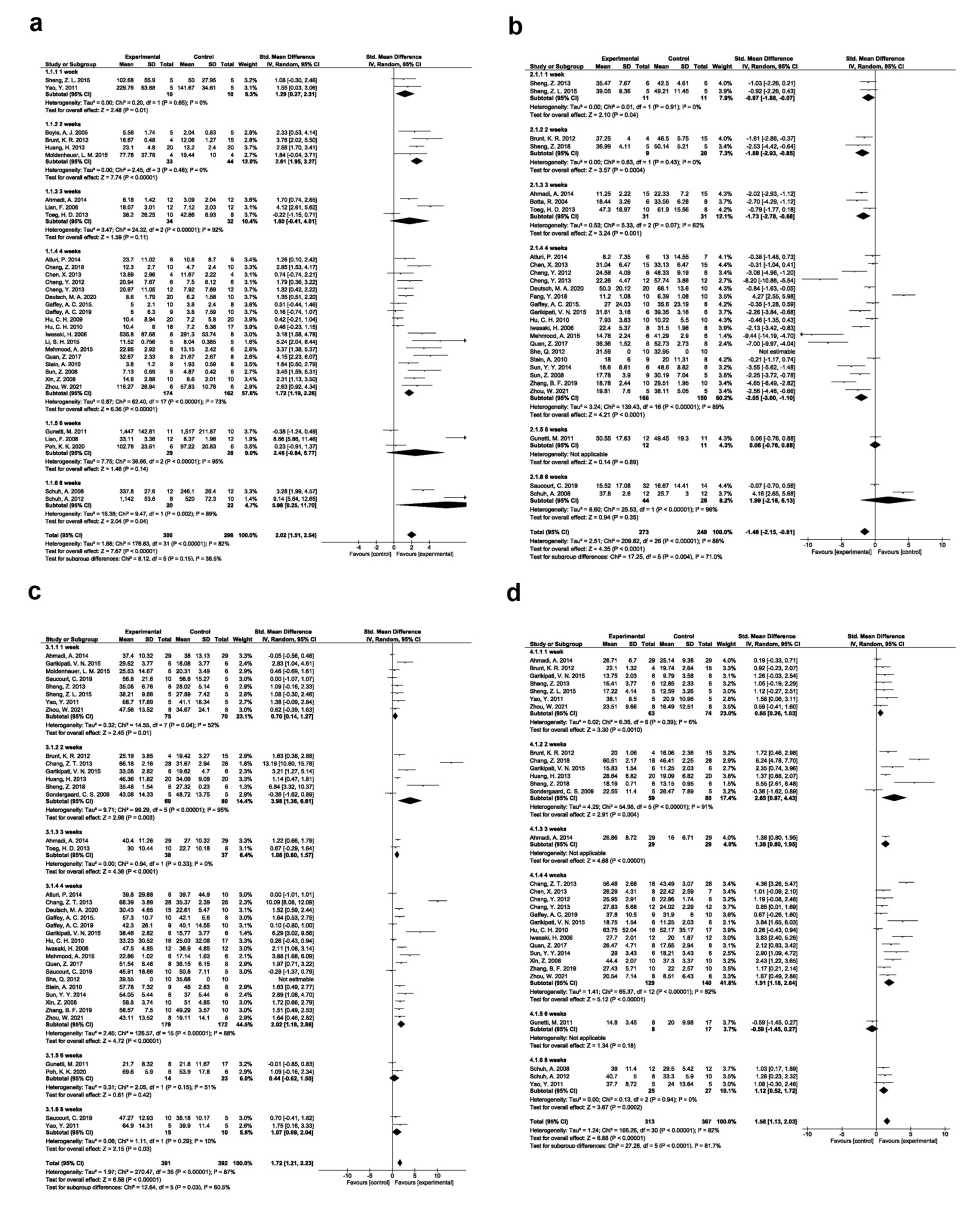
Angiogenesis improvement based on the time of results assessment (a). Fibrosis improvement based on the time of results assessment (b). Ejection fraction improvement based on the time of results assessment (c). Fractional shortening improvement based on the time of results assessment (d)

 A random-effects model was applied to find differences in angiogenesis potential in 32 eligible studies (Figure 3a; SMD: 2.02, 95% CI: 1.51-2.54, *P* < 0.00001; I^2^: 82%). The subgroup analysis of EPC injection in different time points (1, 2, 3, 4, 6, and 8) indicated an improved angiogenesis potential after MI induction. Of note, these changes reached statistically significant levels post EPC injection after one week (SMD: 1.29, 95% CI: 0.27-2.31, *P* = 0.01; I^2^: 0%; N = 2), two weeks (SMD: 2.61, 95% CI: 1.95-3.27, *P* < 0.00001; I^2^: 0%; N = 4), four (SMD: 1.72, 95% CI: 1.19-2.26, *P* < 0.00001; I^2^: 73%; N = 18), and eight weeks (SMD: 5.98, 95% CI: 0.25-11.70, *P* = 0.04; I^2^: 89%; N = 2). The other features were not statistically significant compared to the control group.

###  EPC transplantation effect on myocardial fibrosis

 Data confirmed the reduction of myocardial fibrosis in 28 studies after EPC transplantation compared to the control group (SMD: -1.48; 95% CI − 2.15, -0.81; *P* < 0.0001; I^2^: 88%). Subgroup analysis revealed significant differences of post-EPC administration after one week (SMD: -0.97; 95% CI − 1.88, -0.07; *P*-0.04; I^2^: 0%; N = 2), two weeks (SMD: − 1.89; 95% CI − 2.93, -0.85; *P* = 0.0004; I^2^: 0%; N = 2), three weeks (SMD: − 1.73; 95% CI − 2.78, -0.68; *P* = 0.001; I^2^: 62%; N = 3), and four weeks (SMD: -2.05; 95% CI, − 3.00, -1.10; *P* < 0.0001; I^2^: 89%; N = 18) (Figure 3b).

###  EPC transplantation effect on cardiac ejection fraction

 Random-effects model for differences in LVEF values is shown in Figure 3c. Data showed the efficiency of EPC transplantation in the improvement of LVEF after one week (SMD: 0.70; 95% CI − 0.14, 1.27; *P* = 0.01; I^2^: 52%; N = 8), two weeks (SMD: 3.98; 95% CI 1.36, 6.61; *P*= 0.003; I^2^: 95%; N = 6), three weeks (SMD: 1.08; 95% CI 0.60, 1.57; *P* < 0.0001; I^2^: 0%; N = 2), four weeks (SMD: 2.02; 95% CI 1.18, 2.86; *P* < 0.00001; I^2^: 88%; N = 17), and eight weeks (SMD: 1.07; 95% CI 0.09, 2.04; *P* = 0.03; I^2^: 10%; N = 2) compared to the control group. Despite these results, two experiments reported the lack of statistically significant differences in LVEF parameters after 6 weeks post-EPC administration between the control and EPC groups.

###  EPC transplantation effect on cardiac FS

 Data obtained from a random-effects model indicated significant differences in cardiac FS following EPC therapy in rodent models of MI. To be specific, statistically significant differences were found in FS parameter after one week (SMD: 0.65; 95% CI 0.26, 1.03; *P* = 0.0010; I^2^: 6%; N = 7), two weeks (SMD: 2.65; 95% CI 0.87, 4.43; *P* = 0.004; I^2^: 91%; N = 6), four weeks (SMD: 1.91; 95% CI 1.18, 2.64; *P* < 0.00001; I^2^: 82%; N = 13), and eight weeks (SMD: 1.12; 95% CI 0.52, 1.72; *P*= 0.0002; I^2^: 0%; N = 3) in EPC group as compared with the control group (Figure 3d).

###  Different EPC injection approaches

**Figure 4:**
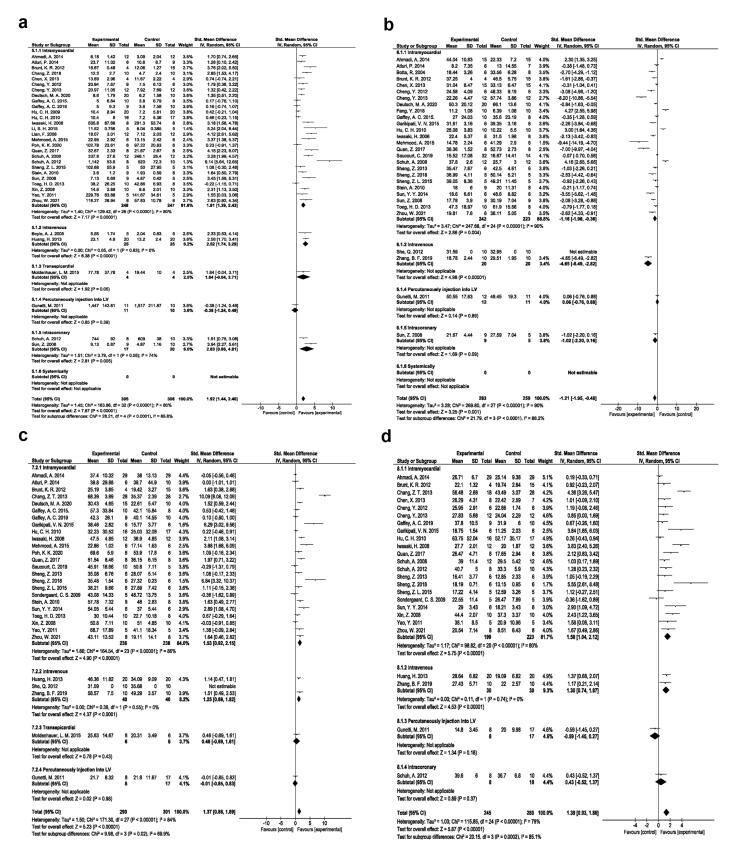
Angiogenesis improvement based on injection method (a). Fibrosis improvement based on injection method (b). Ejection fraction improvement based on injection method (c). Fractional shortening improvement based on injection method (d)

 The regenerative efficacy of the EPC injection route was also assessed in rodent MI models. Intramyocardial route is the commonly used approach for the introduction of EPCs into the ischemic myocardium with the angiogenesis potential (SMD 1.91, 95% CI- 1.39-2.43, *P* < 0.00001, I^2^: 80%; N = 27; Figure 4a); reduction of fibrosis (SMD -1.16, 95% CI- -1.96, -0.36, *P* = 0.004, I^2^: 90%; N = 25; Figure 4b); improving EF (SMD:1.53, 95% CI- 0.92-2.15, *P* < 0.00001, I^2^: 86%; N = 24; Figure 4c), and FS values (SMD:1.58, 95% CI- 1.04-2.12, *P* < 0.00001, I^2^: 80%; N = 21; Figure 4d).

###  Various EPC doses

**Figure 5:**
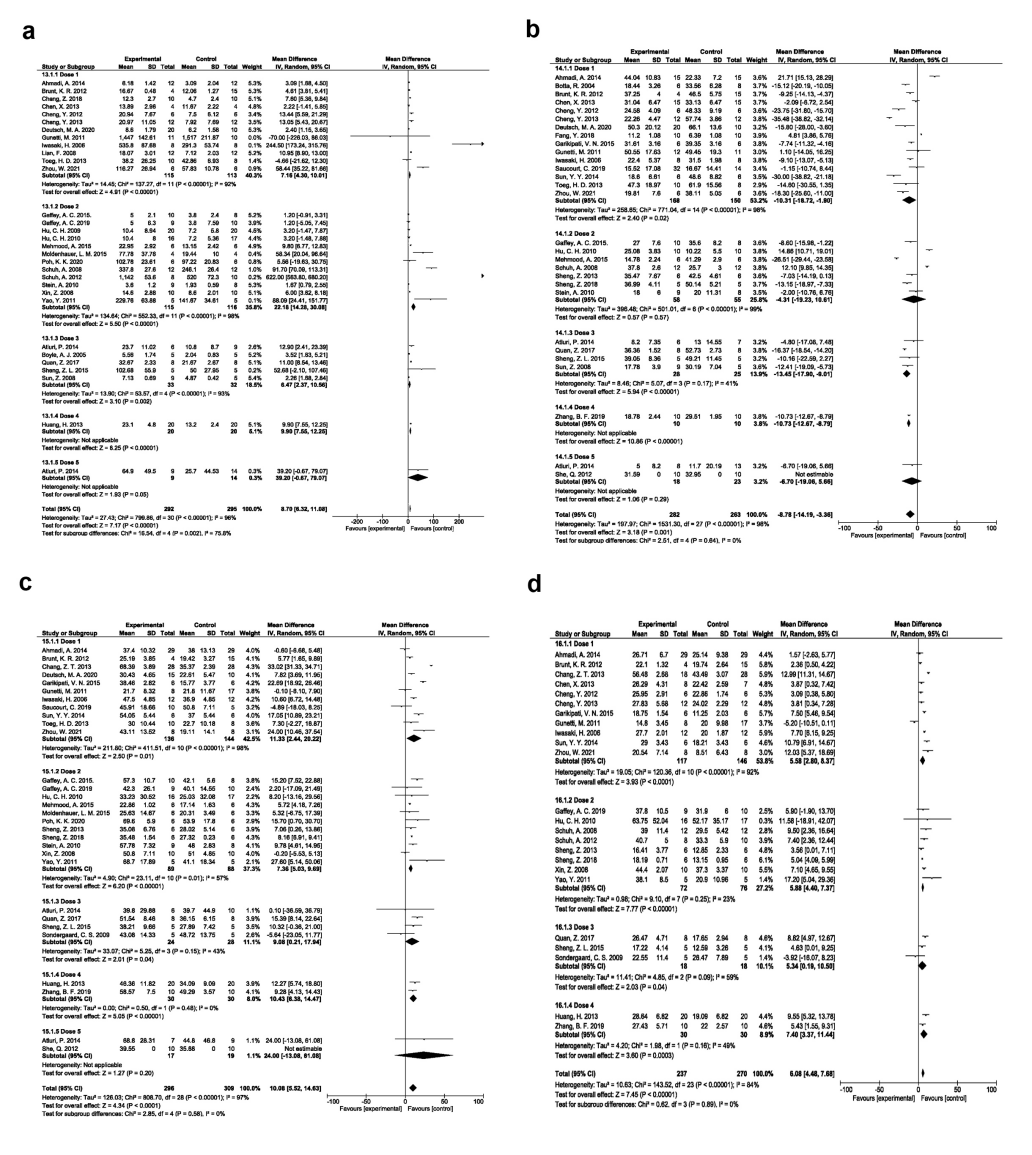
Angiogenesis improvement based on EPC dose (a). Fibrosis improvement based on EPC dose (b). Ejection fraction improvement based on EPC dose (c). Fractional shortening improvement based on EPC dose (d)

 Based on EPC dose, studies were categorized into 5 groups as follows; up to 0.5 × 10^6^, 0.5 to 1 × 10^6^, 1 to 2 × 10^6^, 2 to 5 × 10^6^, and more than 5 × 10^6^ groups. The weighted applied dose to EPC transplantation is dose 1 (up to 0.5 × 10^6^), which demonstrated significant angiogenesis effects (SMD 7.16, 95% CI- 4.30-10.01, *P* < 0.00001, I^2^: 92%; N = 12; Figure 5a), reduced fibrosis (SMD -10.31, 95% CI- -18.72, -1.90, *P* = 0.02, I^2^: 98%; N = 15; Figure 5b), improved EF (SMD 11.33, 95% CI- 2.44-20.22, *P* = 0.01, I^2^: 98%; N = 11; Figure 5c), and FS (SMD 5.58, 95% CI- 2.80-8.37, *P* < 0.0001, I^2^: 92%; N = 11; Figure 5d) compared to the other doses.

###  Various EPC sources

**Figure 6:**
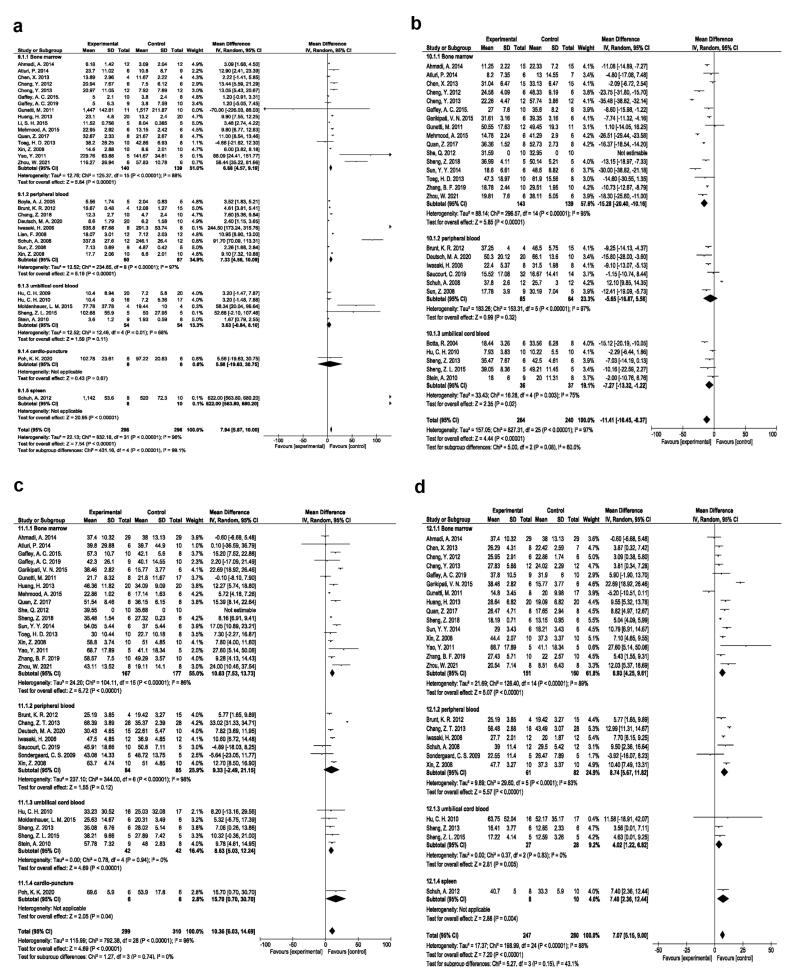
Angiogenesis improvement based on EPC source (a). Fibrosis improvement based on EPC source (b). Ejection fraction improvement based on EPC source (c). Fractional shortening improvement based on EPC source (d)

 Based on our data, it was confirmed that bone marrow EPCs exerted significant angiogenesis effects (SMD 6.88, 95% CI- 4.57-9.19, *P* < 0.00001, I^2^: 88%; N = 16; Figure 6a); reduced fibrosis (SMD -15.28, 95% CI- -20.40, -10.16, *P* < 0.00001, I^2^: 95%; N = 16; Figure 6b); improved EF (SMD:10.63, 95% CI- 7.53-13.73, *P* < 0.00001, I^2^: 86%; N = 17; Figure 6c), and FS (SMD: 6.93, 95% CI- 4.25-9.61, *P* < 0.00001, I^2^: 89%; N = 15; Figure 6d) in comparison with EPC types.

###  Publication bias

**Figure 7:**
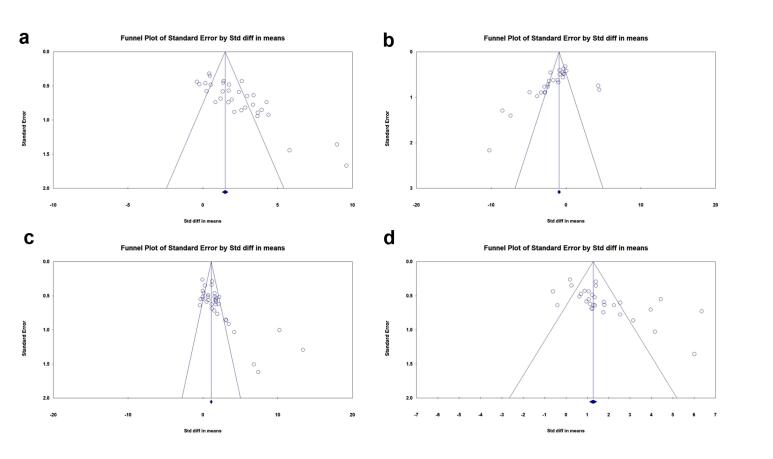
Funnel plot of standard error by the standard difference. Angiogenesis (a); Fibrosis (b); Ejection fraction (c); and Fractional shortening (d)

 Four funnel plots were developed using RevMan 5.4.1 to assess the publication bias among the selected experiments on each outcome (Figures 7a-d). For angiogenesis potential,Begg and Mazumdar’s test revealed Kendall’s tau of 0.546 (z = 4.395, *P* = 0.00001), and Egger’s regression test indicated a significant intercept of 5.79 (SE = 0.855, *P* = 0.00000). For anti-fibrosis properties,Begg and Mazumdar’s test yielded Kendall’s tau of -0.576 (z = 4.211, *P* = 0.00003) with Egger’s regression of -4.62 (SE = 1.407, *P* = 0.00300). In terms of EF,Kendall’s tau of 0.522 (z = 4.481, *P* = 0.00001) was obtained by Begg and Mazumdar’s test, and a noteworthy intercept of 5.43 (SE = 1.011, *P* = 0.00001) was evaluated by Egger’s regression test. Finally, in the FS parameter,Begg and Mazumdar’s test demonstrated Kendall’s tau of 0.460 (z = 3.637, *P* = 0.00028) and an intercept of 4.27 (SE = 1,113, *P* = 0.00062) after Egger’s regression test. These results suggest publication bias based on both the visual inspection of the funnel plot and the statistical tests in all outcomes.

## Discussion

 MI is a debilitating pathological condition with a high rate of mortality in societies.^89^ Therapeutic strategies targeting the increase of vascularization and blood perfusion are beneficial to alleviate the adverse effects of MI. In this regard, in-time blood vessel formation can significantly reduce scar formation, abnormal LV remodeling, and massive cardiomyocyte damage.^90^ Emerging *in vitro*, preclinical, and clinical data have indicated the potency of various stem cell types, especially EPCs, in the restoration of vascularization into the ischemic sites. It was suggested that both maturation into functional ECs, and the release of several proangiogenesis factors can expedite the process of healing in the ischemic sites.^13^ Of note, *in vitro*, *ex vivo* experiments, preclinical studies, and *in silico* analyses are required to evaluate the efficacy and safety of cells or drug candidates before application in the human counterpart.^91^ In this regard, the current systemic review and meta-analysis included preclinical experiments and aimed to explore the effectiveness of EPCs in rodent (rat and mouse) models of MI. Features such as angiogenesis, fibrosis, EF, and SF were monitored in MI animals following the administration of EPCs and compared to the control MI group.

 The present data noted that EPC transplantation can influence primary outcomes such as angiogenesis and fibrosis in MI groups receiving only cell-free phosphate-buffered saline (PBS) or culture medium. Along with these changes, EPC administration led to improvements in cardiac function parameters, such as FS, and EF following MI induction. It has been assumed that several underlying molecular mechanisms are stimulated after the injection of EPCs into ischemic tissues.^92^ For example, EPCs are capable of ensuring cardiac tissue regeneration via the reduction of oxidative stress.^93^ Xue et al found that moderate-to-high doses of EPCs blunt the oxidative stress (8-iso-prostaglandin F2α↓, and SOD↑), and endoplasmic reticulum stress (GRP78 and CHOP) in a rat model of acute MI.^94^ Of course, prolonged exposure to insulting conditions contributes to the induction of oxidative stress in EPCs. Under such conditions, the function of EPCs and angiogenesis potential are fundamentally influenced. Hamed and co-workers found that diabetic circulating EPCs produce higher oxygen free radicals and exhibit higher SOD, NADPH oxidase activity with reduced NO bioavailability compared to normal EPCs.^95^ Therefore, attention should be given to the selection of appropriate EPCs to achieve optimal regenerative outcomes under varying pathological conditions.

 It is hypothesized that direct physical contact between the EPCs and cardiac cells can stimulate several healing processes related to angiogenesis, ECM remodeling, and ventricular function.^12^ Multiple cell death modes such as cardiomyocyte apoptosis, excessive autophagic death, and necrosis are diminished following the administration of EPCs.^96,97^ Besides, EPCs exert anti-fibrotic properties through the modulation of the TGF-β signaling pathway and regulation of Smads.^98^ Of course, the regenerative potential of EPCs is not limited to the above-mentioned mechanisms, and these cells can affect the bioactivity of multiple cardiac cells in a paracrine and juxtacrine manner.^99^ For instance, the EPC secretome contains various signaling factors affecting the function of ECs after injury. In response to the EPC paracrine activity, the angiogenesis potential of ECs is promoted while simultaneously inflammatory damage is reduced in ECs.^100^ One possible explanation for this effect is that the EPC-derived extracellular vesicles harbor high levels of pro-angiogenesis factors, such as VEGF and miR-183, which have the potential to activate the biological activity of ECs at the site of injury.^101^ More interestingly, the differentiation of cardiac cells increases toward endothelial lineage once certain signaling pathways such as Shh are stimulated.^12^ Abd El Aziz et al found that intramyocardial transplantation of 5 × 10^6^ human cord blood EPCs improves cardiac tissue function in a canine model of infarction via localization in the vascular units and direct differentiation into troponin I^+^ cardiomyocytes.^102^ The increase of endothelial nitric oxide synthetase and NO inside ECs is also associated with the paracrine activity of EPCs.^103^ Likewise, both superoxide dismutase and catalase stimulation and the expression of Bcl-2 increase EC resistance to oxidative stress juxtaposed to ischemic myocardium.^12^ Li et al found that shortly after ischemia induction in mice, donor EPCs can rapidly be recruited into the myocardium and elevate the local NO contents via the production of endothelial (eNOS) and inducible nitric oxide synthetase (iNOS).^104^ In line with this, Cristóvão and co-workers indicated lower CD34^+^/KDR^+^ EPC levels in ischemic cardiomyopathy patients compared to healthy counterparts, indicating fast and appropriate recruitment of EPCs in response to hypoxic/ischemic conditions.^105^

 Data have confirmed that the direct juxtacrine activity of EPCs can promote neointima formation via the regulation of pericyte migration, secretion capacity, and phenotypic switching.^106^ Notably, EPCs can be genetically modified before transplantation to increase their regenerative potential.^107^ For instance, miR-214 expressing EPCs efficiently can control calcium hemostasis in stressed cardiomyocytes and enhance survival rate.^12^ Exosomal miR-1246 and miR-1290 driven EPCs upregulate ELF5 and SP1 in cardiac fibroblasts and increase endothelial differentiation.^108^

 In addition to reducing fibrosis, the promotion of angiogenesis, activation of local cardiac progenitor cells, and increase in circulating progenitors within the infarcted myocardium collectively accelerate the healing process.^109^ Therefore, EPC administration appears to promote cardiac tissues through both endogenous and exogenous mechanisms.^110^

 Recent data affirm that the administration route influences the healing capacity and regenerative outcomes by affecting the on-target delivery, stem cell survival rate, and bioactivities.^111^ According to the search we conducted, the direct intramyocardial injection yields better healing properties compared to the other administration routes. The systemic administration could lead to the sequestration of EPCs in certain tissues such as the liver, spleen, and lungs due to massive vascular beds while direct injection into the target tissues provides a higher delivery rate and retention time.^112^ Therefore, the homing of systemically administrated EPCs into the myocardium is less due to low retention time and certain anatomical features of cardiac tissue.^113^ Like intramyocardial injection, the intracoronary EPC infusion is considered to be widely administered. However, this modality requires higher cell volume compared to direct intramyocardial injection. It is worth remembering that the intracoronary route can increase the probability of cell clustering, and embolism, resulting in the occlusion of supporting blood vessels into the affected sites.^114,115^ Although intramyocardial injection ascertains higher cell delivery into the ischemic sites, this approach leads to the loss of a fraction of transplanted cells due to mechanical stress in solid organs such as cardiac tissue. Besides, iatrogenic inflammation and secondary tissue injuries can also occur when the cells are directly administrated into the myocardium.^116^ Like transepicardial and intracoronary routes, the intramyocardial injection essentially requires thoracotomies, which is an invasive surgical approach and cannot be performed when multiple cell doses are required.^117^ Despite the low targeting efficiency of EPC therapy via the systemic route, this approach is suitable for multiple-dose injection purposes.^110,117^ Using special advanced technologies such as ultrasound-guided percutaneous injection, the high cell doses can be directly delivered into different parts of LV in a relatively non-invasive manner. To standardize this approach with minimum side effects, various studies must be conducted

 The statistically significant results of Egger’s and Begg’s tests suggest the possibility of publication bias, implying that studies with statistically significant results may be more likely to be published than studies with null or negative findings. This could lead to an overestimation of the true effect size. Therefore, the results of this meta-analysis should be interpreted with caution. Future research, including studies with negative or null findings, would be valuable to clarify the true effect of EPCs in the restoration of cardiac function following experimentally induced MI in rodents.

 This study has several limitations and future experiments should address them as much as possible. Even though this study made an effort to synthesize the available evidence rigorously, the high heterogeneity observed for most outcomes (I^2^ > 80%) suggests considerable variability between the included studies. Despite the conduction of subgroup analyses, it was not feasible to fully explore the potential sources of this heterogeneity due to limitations in the reported data of the original publications. Due to these features, it was not possible to draw firm conclusions about the specific factors influencing the effectiveness of EPC therapy. In addition, a small sample size related to some parameters would make the interpretation problematic. These limitations highlight the necessity of further experiments to address the gaps and flaws. Specifically, future studies should report detailed data in a more standardized and comprehensive manner in terms of EPC source, dosage, administration route, experimental conditions, and relevant outcome measures.

 The micro-, and microanatomy structure of cardiac tissue and its kinetics profoundly vary in rodents compared to their human counterparts. It is estimated that rodents have high heart rates and short lifespans. Meanwhile, the expression of genes and factors in cardiac cells can in part but not completely differ as compared to the other mammals.^21^ For instance, alpha isoform is the dominant type of myosin heavy chain in humans and large mammals atrium while this protein type is highly expressed in ventricles of mice and rats.^21^ The prominent difference in cardiac tissue kinetics and parameters can lead to relatively incomparable outcomes in rodents receiving stem cells and progenitors compared to large-size mammal models and humans.^118^ EPCs display high similarity with other cell lineages such as hematopoietic stem cells, thus the precise characterization, isolation, and purification of EPCs seem problematic. Besides, EPCs constitute 0.01 to 0.0001% of total bone marrow mononuclear cells, and *in vitro* expansion using different growth factors and supporting ECM components are necessary to yield EPCs in high quantities.^13,119^ Regarding the limited number of EPCs in freshly collected samples, serial passages and prolonged culture time can contribute to the loss of EPC phenotype and functionality.^120^ Although cryopreservation in part preserves the phenotype and biological activity, attention should be given to optimizing the cryopreservation protocols using suitable cryoprotectants to minimize the adverse effects of storage temperature.^121^ Based on the recent data, EPC type and maturation stage can influence the angiogenesis outcomes. Sieveking and co-workers found that later outgrowth EPCs can directly participate in the structure of vascular units better than that of early EPCs. It seems that early EPCs can promote the angiogenesis phenomenon indirectly via the release of angiogenesis factors at the site of injury.^122^ The mobilization of EPCs in response to cytokine gradient increases simultaneous maturation and functional activity compared to the resident progenitors inside the bone marrow niche.^13^ The circulating EPCs can lose their stemness features (CD133↓, and CD34↓) accompanied with the expression of certain markers such as CD31, and vWF with the reaching to the injured site.^13^ These data confirm that bone marrow EPCs are putative progenitor cells in the induction of angiogenesis in the ischemic regions. Besides cell source, the number of graft stem cells can predetermine the angiogenesis outcomes, especially in tissue with chronic injuries. However, less and excessive stem cells can cause the disruption of the healing process via an imbalance in immune cell activity and normal development of resident cells and transplanted stem cells.^123^ Taken together, the number and source of EPCs can be effective in the induction of angiogenesis in the ischemic myocardium.

## Conclusion

 The current systemic review and meta-analysis showed the eligibility of EPCs in the restoration of cardiac function following experimentally induced MI rodents, either rats or mice. The stimulation of angiogenesis and reduction of fibrosis along with the improvement of cardiac functional parameters (EF, and FS) are the main outcomes following EPC transplantation. Taken together, the current data provide new insights into the potential clinical application of EPCs and their regenerative properties in patients with MI.

## Competing Interests

 The authors have declared that no competing interests exist.

## Ethical Approval

 All phases of this study were approved by local ethics committee of Tabriz University of Medical Sciences (ethical code: IR.TBZMED.VCR.REC.1402.198 Approval date: 2023-10-16) and NIMAD (National Institute for Medical Research Development) (ethical code: IR.NIMAD.REC.1402.031; Approval date: 2023-12-30) under research proposal entitled “Application of endothelial progenitor cells in the alleviation of cardiac infarction in rodent models”.

## Supplementary File


Supplementary file contains Table S1 and S2.
