# Supplementary material for: Therapeutic Potential of Endothelial Progenitor Cells in Angiogenesis and Cardiac Regeneration: A Systematic Review and Meta-Analysis of Rodent Models
Source: Adv Pharm Bull. 2025 Jun 16;15(2):268–83. doi: 10.34172/apb.025.45122 (PMC12413962; doi:10.34172/apb.025.45122)
Supplement: Supplementary file 1 — Supplementary file contains Table S1 and S2. [file apb-15-268-s001.pdf]

Adv Pharm Bull. 2025;15(2):268-S1
doi: 10.34172/apb.025.45122
https://apb.tbzmed.ac.ir

Supplementary Table 1. Detailed search strategy

Cochrane

ID	Search
#1	MeSH descriptor: [Myocardial Infarction] explode all trees
#2	(Myocardial Infarct*):ti,ab,kw OR (Cardiovascular Stroke*):ti,ab,kw OR (Heart Attack*):ti,ab,kw OR (Cardiac Infarct*):ti,ab,kw OR (Heart Infarct*):ti,ab,kw (Word variations have been searched)
#3	#1 OR #2
#4	MeSH descriptor: [Endothelial Progenitor Cells] explode all trees
#5	(Endothelial Progenitor Cell*):ti,ab,kw (Word variations have been searched)
#6	#4 OR #5
#7	#3 AND #6

Embase

#1. 'endothelial progenitor cells'/exp
#2. endothelial:ab,ti AND progenitor:ab,ti AND cell*:ab,ti
#3. #1 OR #2
#4. heart:ab,ti AND infarct*:ab,ti
#5. 'heart infarction'/exp
#6. myocardia:ab,ti AND infarct*:ab,ti
#7. cardiovascular:ab,ti AND stroke*:ab,ti
#8. heart:ab,ti AND attack*:ab,ti
#9. cardiac AND infarct*:ab,ti
#10. #4 OR #5 OR #6 OR #7 OR #8 OR #9
#11. #3 AND #10
#12. #11 AND ('article'/it OR 'conference abstract'/it OR 'conference paper'/it OR 'conference review'/it OR 'review'/it)

PubMed

Search number	Query	Sort By	Filters
3	(((((("Myocardial Infarction"[Mesh]) OR (Myocardial Infarct*[Title/Abstract])) OR (Cardiovascular Stroke*[Title/Abstract])) OR (Heart Attack*[Title/Abstract])) OR (Cardiac Infarct*[Title/Abstract])) OR (Heart Infarct*[Title/Abstract])) AND (("Endothelial Progenitor Cells"[Mesh]) OR (Endothelial Progenitor Cell*[Title/Abstract]))		
2	("Endothelial Progenitor Cells"[Mesh]) OR (Endothelial Progenitor Cell*[Title/Abstract])		
1	((((("Myocardial Infarction"[Mesh]) OR (Myocardial Infarct*[Title/Abstract])) OR (Cardiovascular Stroke*[Title/Abstract])) OR (Heart Attack*[Title/Abstract])) OR (Cardiac Infarct*[Title/Abstract])) OR (Heart Infarct*[Title/Abstract])		

Scopus

4	(TITLE-ABS (endothelial AND progenitor AND cell*)) AND (TITLE-ABS (myocardial AND infarct*) OR TITLE-ABS (cardiovascular AND stroke*) OR TITLE-ABS (heart AND attack*) OR TITLE-ABS (cardiac AND infarct*) OR TITLE-ABS (heart AND infarct*)) AND (LIMIT-TO (DOCTYPE , "ar") OR LIMIT-TO (DOCTYPE , "re") OR LIMIT-TO (DOCTYPE , "cp")) AND (LIMIT-TO (SRCTYPE , "j") OR LIMIT-TO (SRCTYPE , "p"))
3	(TITLE-ABS (endothelial AND progenitor AND cell*)) AND (TITLE-ABS (myocardial AND infarct*) OR TITLE-ABS (cardiovascular AND stroke*) OR TITLE-ABS (heart AND attack*) OR TITLE-ABS (cardiac AND infarct*) OR TITLE-ABS (heart AND infarct*))
2	TITLE-ABS (myocardial AND infarct*) OR TITLE-ABS (cardiovascular AND stroke*) OR TITLE-ABS (heart AND attack*) OR TITLE-ABS (cardiac AND infarct*) OR TITLE-ABS (heart AND infarct*)

1 TITLE-ABS (endothelial AND progenitor AND cell*)

Web Of Science

1. Myocardial Infarct* (Topic) or Cardiovascular Stroke* (Topic) or Cardiac Infarct* (Topic) or Heart Infarct* (Topic) or Heart Attack* (Topic)
2. "Endothelial Progenitor Cell" (Topic) or "Endothelial Progenitor Cells" (Topic)
3. #1 AND #2
4. #1 AND #2 and Article or Review Article or Proceeding Paper (Document Types)

Supplementary Table 2. Statistical data of rat and mouse strains included in this study.

Species					
**Rat**	37	55.22%	**Mice**	30	44.78%
Wistar rat	10	27.03%	C57BL/6J	11	36.67%
Sprague-Dawley rat	14	37.84%	Nude	5	16.67%
Athymic nude rat	10	27.03%	BALB/C	5	16.67%
Lewis rat	1	2.70%	BALB/C nude	1	3.33%
Zucker rat	1	2.70%	Sprague-Dawley	1	3.33%
CBH-Rnu rat	1	2.70%	Nude/J or NOD-SCID	1	3.33%
			NOD-SCID	3	10.00%
			Mice (ND)	3	10.00%
